# Automated Reporter Quantification *In Vivo*: High-Throughput Screening Method for Reporter-Based Assays in Zebrafish

**DOI:** 10.1371/journal.pone.0029916

**Published:** 2012-01-04

**Authors:** Steven L. Walker, Junko Ariga, Jonathan R. Mathias, Veena Coothankandaswamy, Xiayang Xie, Martin Distel, Reinhard W. Köster, Michael J. Parsons, Kapil N. Bhalla, Meera T. Saxena, Jeff S. Mumm

**Affiliations:** 1 Department of Cellular Biology and Anatomy, Georgia Health Sciences University, Augusta, Georgia, United States of America; 2 Luminomics, Inc., Augusta, Georgia, United States of America; 3 Cancer Center, Georgia Health Sciences University, Augusta, Georgia, United States of America; 4 Institute of Developmental Genetics, Helmholtz Zentrum München, Neuherberg, Germany; 5 Department of Surgery, Johns Hopkins University, Baltimore, Maryland, United States of America; Center for Regenerative Therapies Dresden Group Leader CRTD, Germany

## Abstract

Reporter-based assays underlie many high-throughput screening (HTS) platforms, but most are limited to *in vitro* applications. Here, we report a simple whole-organism HTS method for quantifying changes in reporter intensity in individual zebrafish over time termed, Automated Reporter Quantification *in vivo* (ARQiv). ARQiv differs from current “high-content” (e.g., confocal imaging-based) whole-organism screening technologies by providing a purely quantitative data acquisition approach that affords marked improvements in throughput. ARQiv uses a fluorescence microplate reader with specific detection functionalities necessary for robust quantification of reporter signals *in vivo*. This approach is: 1) Rapid; achieving true HTS capacities (i.e., >50,000 units per day), 2) Reproducible; attaining HTS-compatible assay quality (i.e., Z'-factors of ≥0.5), and 3) Flexible; amenable to nearly any reporter-based assay in zebrafish embryos, larvae, or juveniles. ARQiv is used here to quantify changes in: 1) Cell number; loss and regeneration of two different fluorescently tagged cell types (pancreatic beta cells and rod photoreceptors), 2) Cell signaling; relative activity of a transgenic Notch-signaling reporter, and 3) Cell metabolism; accumulation of reactive oxygen species. In summary, ARQiv is a versatile and readily accessible approach facilitating evaluation of genetic and/or chemical manipulations in living zebrafish that complements current “high-content” whole-organism screening methods by providing a first-tier i*n vivo* HTS drug discovery platform.

## Introduction

Reporter-based assays have revolutionized the analysis of biological phenomena [1], [2] and increased the pace of drug discovery by facilitating high-throughput screening (HTS) [3]. Such assays typically involve either simple quantitative outputs (e.g., relative reporter units) or “high-content” imaging data (e.g., automated confocal microscopy providing cellular resolution). Automated whole-organism imaging methods have been developed [4], [5], [6], [7] which facilitate the use of zebrafish for high-content phenotype-based screening [8]. Although extremely powerful, such methods are typically limited to mid-throughput capacities (e.g., 5,000 units per day) due to acquisition time and/or data processing limitations. Moreover, broad implementation is hindered by general availability and/or economic issues. To overcome these barriers, we have developed a versatile and readily accessible quantitative *in vivo* screening method that is capable of achieving true HTS capacities.

Interest in performing small molecule screens directly in animal models is increasing in both pharmaceutical and academic research communities [8]. Zebrafish are a well-established vertebrate model system for large-scale phenotypic drug screening due to their small size, rapid external development, and optical transparency [9], [10]. Transgenic zebrafish provide reporter-based read outs for specific cell types, major signaling pathways (e.g., Wnt, Notch, Hedgehog), and other cellular events (e.g. neuronal activity, programmed cell death). Using such resources, automated high-content imaging screens have been used to identify compounds that alter heart rate [11], [12], [13], angiogenesis [7], [14], stem cell specification [15], [16], and even to ameliorate complex dysmorphic syndromes [17]. Conservation of drug effects between fish and mammals has been verified extensively, for instance 22 of 23 drugs linked to QT prolongation in humans similarly altered heart rates in zebrafish [13], [18]. However, as noted above, high-content imaging-based approaches remain limited with regard to throughput and general availability. Moreover, high-content assays using static end points such as antibody staining do not fully account for pharmacodynamics. We reasoned that adapting reporter-based zebrafish assays to simpler quantitative HTS screening technologies could improve throughput and accessibility issues. In addition, we sought to develop methods that would allow changes in reporter signal to be quantified over time scales ranging from minutes to days, thus accounting for disease progression and/or drug action kinetics.

Here, we report a simple HTS method termed Automated Reporter Quantification*i* *n vivo* (ARQiv). ARQiv employs a microplate reader outfitted with specific detection functions that allow changes in fluorescent reporters to be accurately monitored in individual zebrafish over time. Microplate reader detection of fluorescent dyes [19] and bioluminescence of transplanted cells [20] in zebrafish embryos has been previously reported. Here, a microplate reader is used to detect changes in the expression of transgenic fluorescent reporters in living zebrafish embryos, larvae, and juveniles. This work expands the palette of plate reader-based zebrafish assays to include an increasingly sophisticated library of transgenic resources, increases the range of HTS-compatible ages, and demonstrates advantages afforded by time lapse detection of reporters. ARQiv thus provides an *in vivo* preclinical screening platform that complements high-content imaging approaches. A main advantage of using a quantitative versus high-content screening platform is the increase in throughput. In theory, a single plate reader is capable of screening greater than 200,000 subjects per day, however, it should be noted that this assumes a single time point assay in pre-hatch embryos, and HTS automation for arraying this number of embryos in multiwell formats (see Discussion for practical limitations on throughput). Another advantage stems from the fidelity of detection; Z- and Z'-factor statistical analyses (modified to account for internally normalized data, see Methods) show that ARQiv assay ‘quality’ is consistent with HTS demands. Finally, ARQiv is versatile, being adaptable to a range of fluorophores, useful for screening zebrafish from embryonic to juvenile stages, and capable of longitudinal assays that track changes in reporter levels over time. Three examples of the types of assays that are possible with this screening platform are provided, including detection of changes in: 1) Cell number; the loss and regeneration of fluorescently tagged cell types (pancreatic beta cells and rod photoreceptors), 2) Cell signaling; relative activity of a Notch-signaling reporter line, and 3) Cell metabolism; accumulation of reactive oxygen species. Our studies demonstrate that ARQiv facilitates time-resolved, reporter-based assays in living zebrafish at screening volumes that achieve true HTS capacities using readily accessible instrumentation.

## Materials and Methods

### Ethics Statement

This study was carried out in strict accordance with the recommendations in the Guide for the Care and Use of Laboratory Animals of the National Institutes of Health. The protocol was approved by the Institutional Animal Care and Use Committee (IACUC) of Georgia Health Sciences University (Approval Identification Number: BR10-12-391). This institution has an Animal Welfare Assurance on file in the Office of Laboratory Animal Welfare (Assurance Number: A3307-01). Using approved anesthetics (see below), all efforts were made to minimize discomfort and suffering during the experimental procedures.

### Zebrafish Husbandry and Transgenic Lines

Zebrafish were maintained using standardized temperature (28.5°C) and light cycle conditions (14 hours on, 10 hours off) in standard growth media supplemented with paramecia and micron flake (SERA) starting at 5 days post-fertilization (dpf). Previously described transgenic zebrafish strains [21], [22], [23], [24], [25], [26], [27], [28], [29], [30], [31], [32], [33] used in this study include: *Tg(pax6-DF4:gap43-CFP)^q01^*, *Tg(ins:hmgb1-EGFP)^jh10^*, *Tg(Tp1glob:EGFP)^um14^, Tg(pou4f3:gap43-GFP)^s356t^*, *Tg(gfap:GFP)^mi2001^, Tg(nyx:Gal4-VP16)^q16a^; Tg(UAS:gap43-YFP)^q16b^*, *Tg(sox10:mRFP)^vu234^*, *Tg(-2.0omp:lyn-mRFP)^rw035^*, *Tg(UAS-E1b:nfsB-mCherry)^c264^*, *Tg(Tp1glob:hmgb1-mCherry*)*^jh11^, Tg(hsp70l:Gal4)^1.5kca4^, Tg(UAS:myc-Notch1a-intra)^kca3^,* and *Tg(ins:nfsB-mCherry)^jh4^, Tg(nestin:GFP)^zf168^*. The following lines will be described in detail elsewhere: *Et(fos:KalTA4, UAS:nfsB, UAS:gap43-YFP)^gmc700^*, *Et(fos:KalTA4, 5xUAS:nfsB, 14xUAS:gap43-YFP)^gmc701^*, *Tg(-3.7rho:YFP-nfsB)^gmc500^, Tg(ins:PhiYFP-dest1-T2A-nfsB, sst2:tagRFP)^lmc01^.*

### Fluorescence Microplate Reader Assays

**Figure 1:**
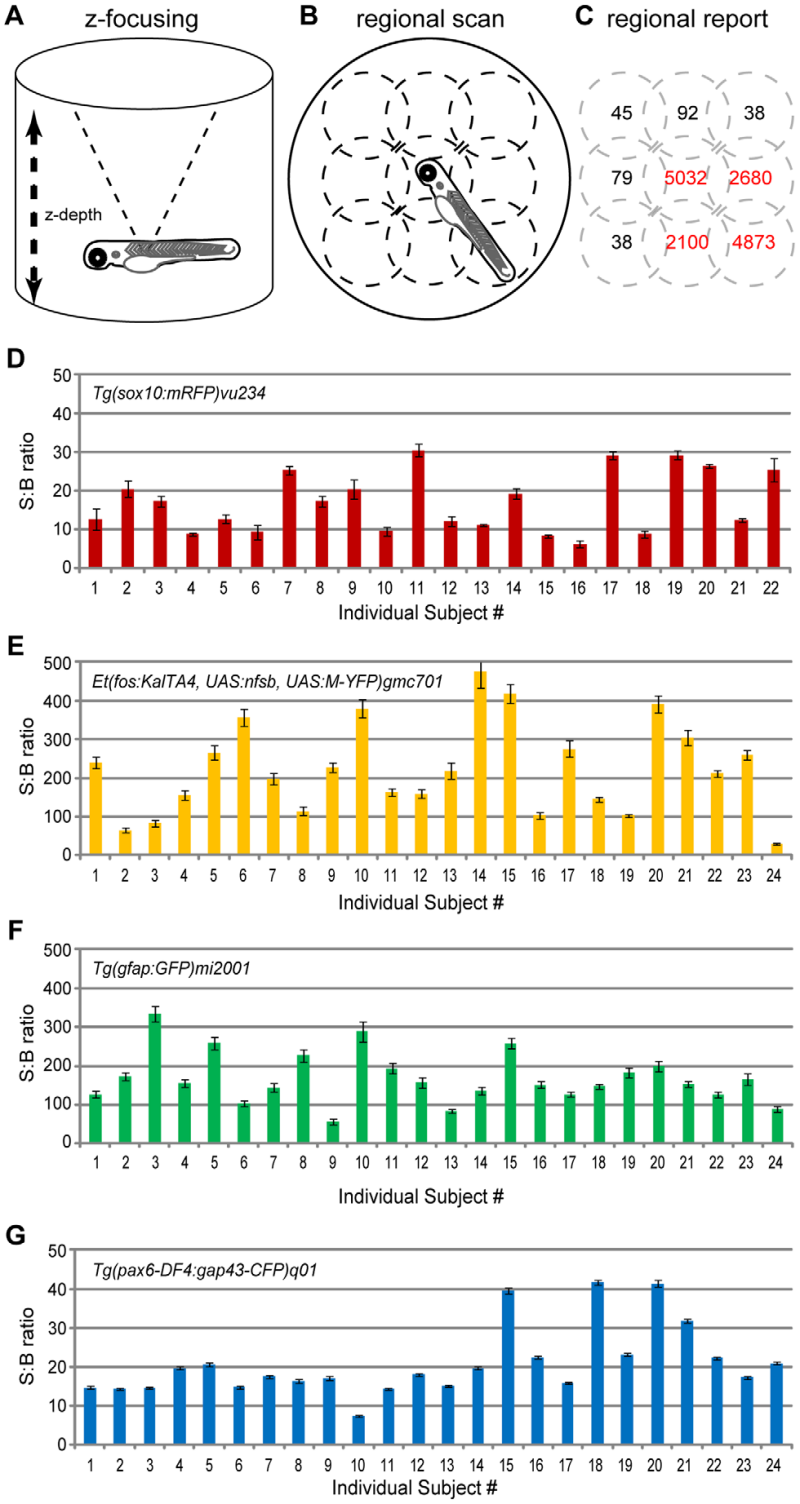
Signal variance across individuals and between fluorphores. Schematics of the scan functions used to detect fluorescent reporters in transgenic zebrafish. A) Z-focus accounts for alternate well shape depth (e.g. conical) and organisms positioned above the nadir of the well. B) Regional scanning accounts for variations in orientations – shown is a 3×3 scan pattern (dotted line circles). C) Regionalized reporting serves to eliminate Background from Signal calculations (i.e., only regions in red are summed to calculate Total Signal). D–G) The fidelity of ARQiv detection was tested using 2 dpf hemizygous embryos of the indicated transgenic lines. Five full scanning sessions – each consisting of three independent scans averaged – were performed over the course of two hours. The resultant Signal to Background (S:B) ratio averages (± standard deviation) across scans per subject indicate that ARQiv detection of fluorescent reporters is highly reproducible. However, despite consistent detection per each individual, a high degree of variability across individuals and across transgenic lines is evident. At the extreme, this difference could be as much as an order of magnitude between sibling clutchmates (compare #14 to #24 in E). This finding indicates that accurate detection of changes in report expression requires signal levels to be normalized per each individual, optimally to pre-manipulation scan readings (see text for further details).

**Figure 2:**
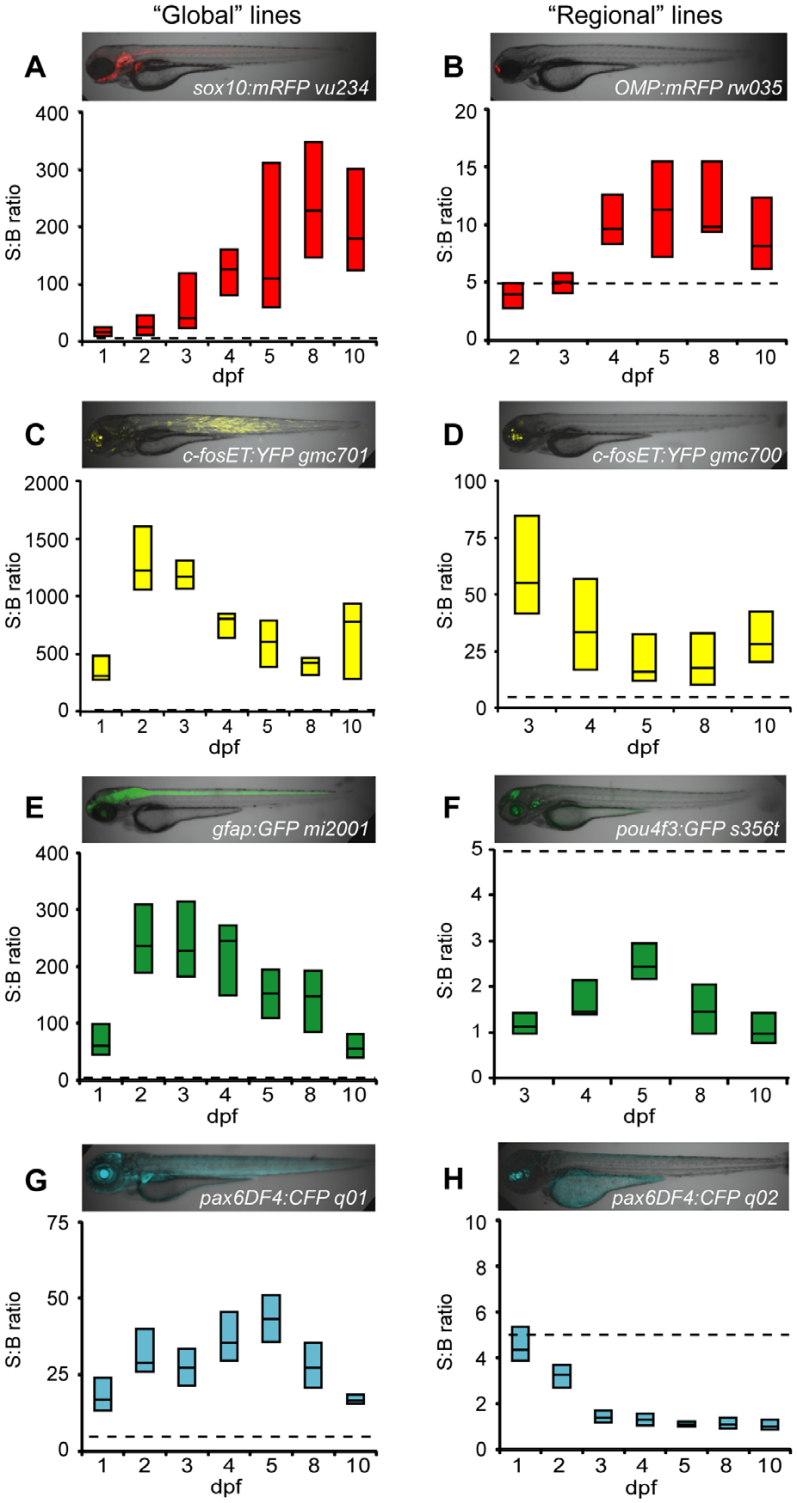
Comparison of fluorescent reporters. Box plots of S:B ratios of transgenic lines expressing different fluorescent reporters across time: A–B) mRFP; C–D) EYFP; E–F) EGFP; G–H) ECFP. Micrographs show the expression patterns of each line. ARQiv scans were performed starting from the earliest day reporter could be detected through 10 dpf. All ‘global’ expressing lines (A,C,E,G) had S:B ratios consistent with excellent HTS assay quality (Z'-factor≥0.5 across ages tested, see Table 3). In contrast, only “red” fluorophore (B) and YFP (D) expressing “regional” lines produced S:B ratios consistent with HTS (see Table 3). Box plots: bottom line is the 25th percentile, top line the 75th percentile, interface between boxes represents the median (n = 8–24 subjects per line). Note: y-axis ranges differ markedly, dashed line indicates 5∶1 ratio of S:B for comparisons of levels across transgenic lines.

Initial attempts at using microplate readers to quantify fluorescence reporter levels in zebrafish proved ineffective. After testing numerous models we identified two screening functionalities critical for whole-organism reporter detection: 1) user- or signal-definable z-dimension focus (Fig. 1A) and 2) the capacity to detect and report measurements on a region by region basis per well (Fig. 1B–C, see text for details). In addition, we found that the only scan mode capable of reproducible *in vivo* reporter detection was the top read. Finally, to promote facile detection of a variety of fluorophores we favored monochromator-based detection models. Accordingly, a microplate reader having all of these functions (Tecan Infinite M1000) was used for all ARQiv assays.

For scans, subjects were individually arrayed in black polypropylene microwell plates having either V-shaped (pre-hatching) or U-shaped (post-hatching) wells. Of the microplates tested, Greiner (#651 209, #650 209, and #655 209) had the lowest autofluorescence profile and provided the highest Signal to Background (S:B) ratios – U-shaped well plates (#650 20) proved optimal across all ages tested (see Table 1 for a list of optimized scan parameters). To further promote reporter detection, most transgenic lines were crossed into pigmentation mutant backgrounds [e.g., *roy orbison* (*roy*), or *roy orbison*; *albino* (“*ruby*”) [34]; *roy* and wildtype fish were also treated with the tyrosinase inhibitor 1-phenyl-2-thiourea (200 nM PTU; Sigma) to reduce melanin synthesis starting at 16–24 hours post-fertilization (hpf) onwards. During scans, fish were anesthetized in tricaine methansulfonate (160 ng/ml, Argent) or Eugenol (50 ng/ml, Sigma). Motility assay tests determined that zebrafish anesthetized with Eugenol are less prone to light-induced startle movements (data not shown). Further testing determined that Eugenol facilitates more consistent readings across successive scans and between individual scan flashes. Finally, we noted that Eugenol promoted improved viability over successive anesthetic treatments compared to Tricaine. For these reasons, we favor the use of Eugenol for ARQiv assays. Gain and z-focus settings were obtained from representative wells. Z'-factor analysis was used to establish data acquisition and processing methods that maximized throughput and reproducibility (see Tables 1, 2, and 3). Fish were removed from anesthetic immediately after scans were complete and maintained in standard growth media. For time-resolved assays, fish were individually re-arrayed in clear multiwell plates allowing general health to be monitored until the next scanning session.

**Table 1:** Recommended acquisition parameters.

Parameter	Optimal
Well Shape*	U-bottom
Volume (Greiner U-bottom 96 well)**	>300 µL
Scan Pattern+	2×2
Flash Mode#	1 (400 Hz)
Number of Flashes	5

*Promotes hands-free centering of subjects.

**Filling the well to eliminate the meniscus effect (∼340 µL) produced the best results, however, this practice proved impractical for HTS.

+ Promotes maximal throughput and consistently high Z'-factors (Table 2 & 3).

# Lethality issues were evident at the 400 hertz level when volume was ≤100 µL.

**Table 2:** Parameters impacting throughput.

Scan pattern	single	2×2	3×3	4×4	5×5
Z'-factor Averages	0.67	0.77	0.79	0.76	0.78
Scan Time (96 well per scan)	00∶26	1∶34	2∶49	3∶40	8∶37
Scan Time per subject (96 well)	0.27 sec	0.94 sec	1.76 sec	2.29 sec	5.39 sec
Estimated daily capacity per reader*	>200,000	>75,000	>45,000	>35,000	>15,000
**Number of Flashes** **	**2**	**5**	**10**	**20**	**50**
Z'-factor Averages	0.71	0.85	0.86	0.87	0.87
Scan Time (96 well per scan)	00∶23	0∶26	0∶31	0∶41	1∶13
Scan Time per subject (96 well)	0.24 sec	0.27 sec	0.32 sec	0.43 sec	0.76 sec
Estimated daily capacity per reader	>215,000	>200,000	>180,000	>140,000	>90,000

Constants: Settle time = 40 ms, Limited to green, yellow and red fluorophore expressing lines.

*Calculated by adding 15 seconds robotics handling time per 96 well plate.

**Single scan pattern used for all calculations.

**Table 3:** Scan pattern Z'factors.

Lines (*allele*) tested*	Automation**	Scan Pattern						
		single	2×2	3×3	4×4	5×5	6×6	7×7
*q01* (global)	Oriented	0.86	0.91	0.86	0.90	0.81	0.78	0.75
	Automated	0.85	0.90	0.85	0.89	0.80	0.80	0.80
*q02* (regional)	Oriented	−16.7	−10.8	−14.6	−8.0	−11.0	−11.0	−7.0
	Automated	−1.1	−3.16	−2.0	−8.0	−30.0	−6.0	−11.0
*mi2001* (global)	Oriented	0.34	0.85	0.86	0.91	0.88	0.85	0.88
	Automated	0.41	0.77	0.76	0.83	0.89	0.87	0.90
*s356t* (regional)	Oriented	0.46	−0.97	−1.8	−8.6	0.17	0.51	0.56
	Automated	−6.6	−7.1	−21.0	−4.3	−1.2	−0.19	−0.89
*gmc701* (global)	Oriented	0.85	0.88	0.91	0.81	0.85	0.85	0.86
	Automated	0.66	0.74	0.84	0.84	0.87	0.83	0.83
*q16a; q16b* (regional)	Oriented	0.75	0.87	0.84	0.82	0.85	0.83	0.82
	Automated	0.78	0.78	0.64	0.62	0.73	0.79	0.84
*vu234* (global)	Oriented	0.87	0.88	0.85	0.86	0.81	0.82	0.76
	Automated	0.66	0.84	0.85	0.86	0.80	0.82	0.85
*q16a; c264* (regional)	Oriented	0.32	0.69	0.70	0.64	0.64	0.74	0.72
	Automated	0.49	0.51	0.67	0.63	0.66	0.69	0.75

*Lines were evaluated at 3 dpf or 6 dpf – i.e., once transgene expression was robust – with comparisons between global and regional lines per each reporter “color”; *q01* and *q02*, CFP, *mi2001* and *s356t*, GFP, *gmc701* and *q16a; q16b*, YFP, *vu234* and *q16a; c264*, RFP. n = 24 to 48 fish per assay.

**Comparison between subjects that were oriented by hand for optimal positioning to those that were dispensed in an automated fashion.

Excitation, emission and respective bandwidth monochromator settings promoting maximal S:B ratios were determined for each fluorophore using 2D spectral scans (in nm): cyan fluorescent protein (CFP, ex444/15, em493/20), green fluorescent protein (GFP, ex485/5, em510/15), yellow fluorescent protein (YFP, ex514/5, em538/10), destabilized monomeric yellow fluorescent protein (PhiYFP-dest1, ex520/5, em538/5), monomeric red fluorescent protein (tagRFP, ex555/5, em585/10), mCherry (ex568/5, em610/10), 2′, 7′-dichlorodihydrofluorescein-diacetate (DCFH-DA, ex488/5, em515/5). These settings also ensure cross-talk between fluorophores is minimal to non-existent, with the exception of GFP and YFP.

For data processing, “Background” was defined as the average plus three standard deviations of maximal regional values from non-fluorescent sibling controls (i.e., the instrument detection limit). “Signal” was defined as any single regional value above background. “Total Signal” was calculated per subject as the sum of all regions producing detectable signal per scan, and averaged across scans in cases where multiple scans were performed. When regional signals dropped below detection limits (i.e., upon cell loss during regeneration assays), one of two methods was applied to calculate Total Signal: 1) when applicable, neighboring control cells expressing a complementary reporter were used to identify regions containing the tissue of interest and corresponding regional values for the experimental fluorophore were summed; 2) in the absence of labeled control cells, maximal regional values were used.

To calculate relative changes in reporter expression over time, initial readings (e.g., pre-treatment) were used to normalize all subsequent scans per each individual subject. Thus, values could be compared across times in relative percentage terms rather than as raw fluorescence values. This approach accounts for variability in reporter expression across individuals (Fig. 1D–G) and allows relative changes in expression to be more accurately quantified across fish. Non-treated transgenic controls were used to establish a correction factor for the relative percentage values reporter for treated fish; i.e., to control for any non-induced fluctuations in reporter expression and/or detection over time. When making comparisons to non-transgenic controls, relative fluorescence values were reported, since absolute signal levels provide a better metric for reporting differences between transgenic and non-transgenic controls.

### Cell Ablation Assays

Transgenic expression of the bacterial enzyme nitroreductase (*nfsB*, NTR) facilitates prodrug-inducible cell-type specific ablation in zebrafish [29], [35]. Transgenic lines expressing NTR and a fluorescent reporter in specific cell types were used to determine whether ARQiv could detect the selective loss and regeneration of fluorescently labeled cells. Prior to cell ablation, NTR-expressing and control non-expressing larvae were scanned to determine pre-treatment fluorescence and background levels. Afterwards, larvae were treated for 24 hrs with media containing the prodrug metronidazole (MTZ, 10 mM for pancreatic beta cell ablation, 2.5 mM for rod photoreceptor cell ablation) to induce ablation of NTR-expressing cells, or with control media. After 24 hrs, all wells were rinsed three times and returned to control media. Subsequent post-treatment and recovery scans were performed at the indicated intervals. Confocal imaging was used to verify the loss and return of fluorescent reporters in the targeted cell types in identically treated subjects.

### Reactive Oxygen Species (ROS) Assays

The compound 2′, 7′-dichlorodihydrofluorescein-diacetate (DCFH-DA, Invitrogen) was used to monitor accumulation of reactive oxygen species in embryonic and larval zebrafish. DCFH-DA is cleaved intracellularly by nonspecific esterases to form DCFH, which is further oxidized by ROS to form the fluorescent compound DCF. 4 hpf embryos were pre-treated with either *N*-acetylcysteine (NAC, 100 µM - a potent ROS scavenging compound which serves as a control for the specificity of the assay) or vehicle control for 2 hrs. Afterwards, embryos were exposed for 30 min to the following known oxidative stressors, hydrogen peroxide (H_2_O_2_, 1 mM), arsenic trioxide (As_2_O_3_, 10 µM), phenethyl isothiocyanate (PEITC, 5 µM). DCFH-DA (50 µM) was then added for 1 hr, and embryos transferred individually to V-shaped microwell plates and subjected to a single post-treatment scan.

### Statistical Analysis

Two commonly used statistical tests to assess HTS assay ‘quality’ (i.e., intersubject variability and dynamic range) are the Z-factors (Z' and Z) [36]. Z-factor values are dimensionless and vary between –∞ and 1 with 0 to 0.5 indicating an assay of acceptable quality, >0.5 signifies excellent quality, while values below 0 indicate assays that should be improved prior to pursuing compound screens. Z'-factor is used to develop and/or optimize HTS assay quality prior to full-scale screens by comparing the mean and standard deviation of positive and negative controls in the absence of test compounds (e.g., transgenic and non-transgenic fish). However, this calculation was designed to account for variation in positive and negative control populations. In our case, we control for the inherent variability of transgene expression across individuals (e.g., Fig. 2) by monitoring changes per each individual and normalizing accordingly. We therefore modified the analysis by calculating Z'-factors per each individual (with standard deviations coming from successive scans rather than across the population) and averaging Z'factors across individuals. In this manner, we were able to assess the capacity of the plate reader to detect and accurately quantify specific transgenic reporters in individual fish (e.g., see Fig. 2). Accordingly, Z'-factor optimization and S:B ratios (calculated as described in the text) were used to define ARQiv parameters promoting optimal assay quality (see Tables 1, 2, and 3).

Z-factor calculations (i.e., not Z') compare the mean and standard deviation of treated samples and a reference control to evaluate assay quality in the presence of test compounds; this determines the capacity of the assay to identify conditions causing significant changes at a single time point (i.e., to define “hits”). In our case, we were interested in determining how well reporter expression changes could be detected over time in individuals (e.g., cell loss and regeneration). We therefore modified these definitions such that ‘reference controls’ were defined as a ‘prior state’ of each subject and ‘treated samples’ defined as a ‘post state’ of the same subject. This takes advantage of the fact that detection of reporters over time in individuals allows all data to be internally normalized; i.e., to calculate relative changes in reporter levels over time, the ‘prior state’ of each fish (e.g., pretreatment) is set at 100% and subsequent changes expressed as a percentage thereof. Thus, to calculate Z-factors ‘post state’ percentages were averaged across fish for each treatment condition at each point of interest (e.g., post-treatment and recovery). Z-factors were calculated for multiple runs of each assay and are reported as the average±sem.

Pairwise comparisons were performed by paired t-test, multiple comparisons by ANOVA, using Excel. Graphs were generated using MATLAB and Excel.

## Results

### Variability of Reporter Expression Across Individuals and Between Transgenic Lines

Two plate reader scanning options facilitate robust reporter detection in living zebrafish: z-dimension focus (Fig. 1A) and regionally-delineated whole well scanning (Fig. 1B–C). The ability to focus in the z-dimension accounts for fish being positioned above the bottom of the well – where most readers are focused and particularly important when using non-standard well configurations (e.g., U-shaped). Regionally defined scans can account for random positioning of subjects within the well and serve to eliminate background from signal calculations (Fig. 1C).

To test reproducibility of detection, hemizygotic transgenic embryos expressing cyan, green, yellow, and red fluorescent reporters (CFP, GFP, YFP, and RFP, respectively) were individually arrayed into conical multiwell plates. At 2 days post-fertilization (dpf) a series of five independent scans were taken over the course of 2 hours. The average Signal to Background (S:B) ratio was then calculated for each individual subject (Fig. 1D–G). The data showed that ARQiv detection of fluorescent reporter levels in individual fish embryos was consistent over repeated scans. However, relative levels across individuals differed by as much as an order of magnitude. Despite reporter variance being a general observation when viewing transgenic siblings under fluorescence microscopy we were surprised that the relative levels could range so dramatically when quantified (see Discussion for possible explanations). This finding has important practical implications; to reduce “noise” from intersubject variability, baseline reporter levels need to be established for each individual subject prior to manipulations. All subsequent scans can then be expressed as a percentage of baseline rather than as raw values, promoting comparisons of relative changes across populations, and therefore across experimental conditions. This approach was used for all time-resolved comparative assays reported here.

### Autofluorescence and Optimal Fluorescent Reporter Evaluation

Large S:B differences were noted when comparing across different transgenic lines (e.g., note differences in y-axis scale in Fig. 1D–G). This could either be due to disparities in expression levels of the lines assayed or to differences in signal to background of reporter variants (e.g., higher autofluorescence background – endogenous fluorescence from internal tissues – at particular wavelengths). We were interested in determining whether specific reporters provided better S:B detection. We reasoned that optimal reporters would be those whose emission spectra produced minimal autofluorescence. Autofluorescence was, therefore, evaluated across a range of ages, pigmentation backgrounds, and in the presence of the pigmentation blocking agent 1-phenyl-thiourea (PTU). Confocal images taken from 3–7 dpf determined that the yolk produced the most prominent autofluorescence, strongest in the blue to green emission ranges, but largely absent from the yellow to magenta (far-red) range (data not shown). ARQiv autofluorescence scans performed from 1–5 dpf were consistent with imaging results but also showed that reductions in pigmentation, regardless of method (i.e., PTU or mutation), resulted in marked increases in autofluorescence detection (Fig. S1). Nevertheless, pigmentation from melanophores and iridophores diminishes the capacity to detect reporter signals (data not shown). Thus, despite the increase in autofluorescence detection, accurate quantification of fluorescent reporters requires methods to reduce pigmentation. Together, imaging and ARQiv data suggests that fluorophores which emit in the longer wavelength ranges may provide better signal detection. However, further analysis showed that autofluorescence changes with age; at adult stages “red” autofluorescence becomes prominent in the yolk/gut region (Fig. S2). This result highlights the need to consider autofluorescence on an age- and/or tissue-specific basis to select a reporter fluorophore having optimal S:B properties.

Next, we sought to determine which fluorescent reporter protein(s) provided the strongest S:B ratios in embryos and larvae – reasoning that younger ages are most relevant to HTS. Optimal excitation/emission wavelengths and bandwidths were established for each fluorophore tested (see Materials and Methods). Two transgenic lines were evaluated per fluorophore (CFP, GFP, YFP or RFP), one having strong reporter expression throughout the body (‘global’ lines), the other being restricted to the head (‘regional’ lines; see micrograph insets in Fig. 2). This allowed us to determine what effect the relative level and/or localization of reporters had on signal detection. ARQiv readings were taken from the earliest day reporters could be detected through 10 dpf. Box plots indicate the range of variability in S:B ratios within and across populations (Fig. 2). The analysis showed that all fluorophores were easily detected when expressed “globally” (Fig. 2A, C, E, and G). Moreover, all global lines met or exceeded ‘assay quality’ levels necessary for HTS (see below). Conversely, only the YFP-expressing “regional” line produced S:B ratios consistent with HTS demands (Fig. 2D, compare to Fig. 2B, F, and H). As predicted by autofluorescence analyses, YFP provided excellent signal detection (Fig. 2C–D, note increased y-axis range). However, autofluorescence data also suggest that red and infrared fluorophores could be useful as well. Accordingly, we investigated whether alternatives to mRFP (a fluorophore known to have suboptimal properties) could improve ARQiv detection. Transgenic lines were established expressing tagRFP and mCherry in subsets of cells in the pancreas and retina, respectively [*Tg*(*ins:PhiYFP-2A-nfsB, sst2:tagRFP)^lmc01^* and *Tg*(*nyx:Gal4-VP16)^q16a^; Tg(UAS:gap43-YFP)^q16b^; Tg(UAS:nfsB-mCherry)^c264^*]. Analyses with these ‘red’ range fluorophore lines showed that tagRFP and mCherry reporters provided improved S:B ratios that are consistent with HTS (e.g., average of 87∶1 for both). Nonetheless, a direct comparison of YFP and mCherry or tagRFP in these lines verified that YFP provides superior signal detection (S:B average of 101∶1). Collectively, the data show that fluorophores with emission peaks of ≥525 nm can provide HTS-compatible signal detection. Having identified an optimal fluorophore range for embryonic and larval screens, we next sought to establish scanning practices which optimized assay ‘quality’ while maintaining high-throughput capacities.

### ARQiv Assay Quality Optimization (Z'-factor Analysis)

Adapting an assay to large-scale screening volumes necessary for HTS involves reducing detection variability and maximizing S:B ratios. A simple statistical coefficient, the ‘Z’-factor', which accounts for variability in sample detection (i.e., positive controls), background signals (i.e., negative controls) and the dynamic range of the assay, was developed to assess assay ‘quality’ [36]. Z'-factor (and Z-factor, see below) values are dimensionless and vary between –∞ and 1. Values that are >0.5 signify excellent assay quality, values from 0 to 0.5 indicate acceptable quality, a value of 0 is limited to binary (yes/no) assays, and values below 0 indicate assays that should be improved prior to pursuing high throughput compound screens.

Z'-factors and/or analysis of variance (ANOVA) on S:B calculations were used to establish ARQiv acquisition parameters that optimized performance. Plate reader parameters were systematically varied and scans repeated five times per condition to establish acquisition conditions that maintained HTS-compatible assay quality while maximizing throughput (Tables 1, 2, and 3). Media volume had significant effects with completely filled wells providing optimal assay quality, presumably due to elimination of the meniscus effect. Parameters effecting throughput – e.g., number of excitation flashes, settle time, scan pattern - could be markedly reduced without significant loss in assay quality (Tables 2 and 3). There was a general trend toward higher Z'-factors with increased “repeats” (i.e., 20 flashes versus 2). However, these assays indicated a 2×2 scan pattern and 5 excitation flashes provide near maximal assay quality. Thus, only in cases where assay quality needs to be improved to meet HTS requirements should increasing “repeat” parameters be used to improve performance, as this comes at the expense of throughput. Importantly, this analysis determined that scan speeds could reach as fast as 0.27 seconds per embryo (1×1 scan pattern) and 0.94 seconds per larvae (2×2 scan pattern). This is consistent with daily screening volumes necessary to achieve true HTS capacities (e.g., ≥50,000 units per day, see Table 2).

### Quantification of Cell Loss and Regeneration

We have developed a cell-specific inducible ablation methodology to extend the study of regeneration in zebrafish to the level of individual cell types [29], [35], [37] (see also Materials and Methods). We reasoned that a non-subjective method for rapidly and accurately quantifying changes in cell number (e.g., of fluctuations in cell-specific reporter levels) would facilitate efforts to monitor the kinetics of regeneration during large scale screens aimed at identifying genes and compounds which can alter the regenerative process. Accordingly, we tested whether ARQiv could be used to accurately detect the loss and regeneration of fluorescently tagged cellular subtypes in individual zebrafish over time.

**Figure 3:**
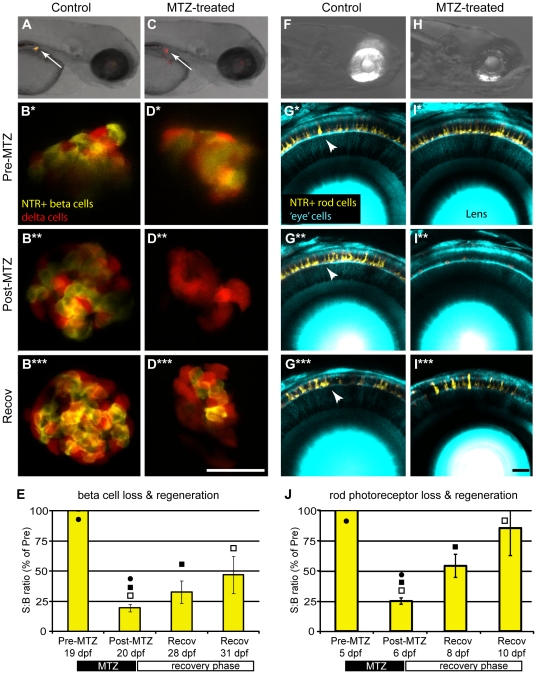
ARQiv detection of cell loss and regeneration. Inducible cell-type specific ablation in nitroreductase (NTR) expressing transgenic fish [29], [35] was used to test whether ARQiv could detect the loss and regeneration of fluorescently tagged cells: A–E) *Tg(ins:PhiYFP-2A-nfsB, sst2:tagRFP)^lmc01^* – targeting pancreatic beta cells, and F–J) Double transgenic *Tg(pax6-DF4:gap43-CFP)^q01^; Tg(-3.7rho:YFP-NTR)^gmc500^* – targeting retinal rod photoreceptors. Beta cell targeting: A) Non-treated control larvae showing PhiYFP-NTR-expressing (i.e., targeted) beta cells (yellow) and neighboring tagRFP-expressing (i.e., non-targeted) delta cells (red) in the pancreas (arrow). B-B^***^ series) Confocal time-lapse imaging shows reporter expression in beta and delta cells is stable in non-treated control larva from pre-treatment (B and B*, 3 dpf) through post-treatment (B^**^, 4 dpf) and recovery (B^***^, 10 dpf). C) MTZ-treated larvae showing loss YFP-NTR-expressing (i.e., targeted) pancreatic beta cells (arrow, absence of yellow). C–D series) Metronidazole (MTZ) treated larvae showing MTZ-induced loss of YFP-NTR-tagged beta cells (absence of yellow; C arrow, and D^**^, 4 dpf) and subsequent regeneration after MTZ wash out and recovery (D^***^, 10 dpf). Unperturbed reporter expression in neighboring tagRFP-tagged delta cells (red; C arrow, and D–D^***^) demonstrates the specificity of the technique. E) ARQiv data demonstrating that beta cell regeneration kinetics can be monitored in late larval to juvenile stage fish (19 to 31 dpf). Paired t-test p-values: • = 0.01, ▪ = 0.33, □ = 0.01. Rod photoreceptor targeting: F) Non-treated control larvae showing YFP-NTR-expressing rod cells (yellow) in eye. G–G^***^) Confocal time-lapse series showing YFP reporter expression in rod cells (arrowheads) is stable over time in non-treated control larva from pre-treatment (G, 5 dpf) through post-treatment (G^*^, 7 dpf) and recovery (G^***^, 10 dpf). H-I series) Metronidazole (MTZ) treated larvae showing MTZ-induced loss of YFP-NTR-tagged rod cells (reduction of yellow in H, and I^**^, 7 dpf) and subsequent regeneration after MTZ wash out and recovery (I^***^, 10 dpf). J) ARQiv data demonstrating that rod cell regeneration kinetics can be monitored in larval to fish (5 to 10 dpf); paired t-test p-values: • = 0.001, ▪ = 0.015, □ = 0.001. Data is presented as mean±standard error. All scale bars are 50 µm.

Two transgenic lines targeting different cellular subtypes – insulin-producing beta cells of the pancreas *Tg(ins:PhiYFP-T2A-nfsB; sst2:tagRFP*)*^lmc01^*, and rod photoreceptors *Tg(-3.7rho:YFP-nfsB)^gmc500^* – were used for this study (both lines will be described in detail elsewhere). Briefly, 995 bp [29], [35], 2.5 kb [38], and 3.7 kb [39] upstream regulatory regions of the *insulin* (*ins*), *somatostatin 2* (*sst2*), and *rhodopsin* (*rho,* kind gift of Dr. Shoji Kawamura) genes were used to restrict expression respectively to pancreatic beta-cells, delta-cells, and rod photoreceptors of the retina. Z'-factor analyses of transgenic positive controls and non-transgenic negative controls showed that ARQiv detection of YFP+ rod cells was compatible with HTS [Z'-factor average of 0.6 ±0.06 (sem)], while detection of beta cells was restricted to binary ‘yes/no’ type assay limits [Z'-factor average −0.1±0.2 (sem)]. For the beta cell line, this likely reflects the low number of cells expressing the reporter or use of a destabilized reporter variant (PhiYFP-dest1, Evrogen). We are currently designing alternative reporter strategies in an effort to improve detection. Another option to overcome this issue is to simply eliminate low expressing individuals using automated fluorescence activated sorting when arraying fish into multiwell formats (see Discussion). All cell regeneration experiments were performed using the same general protocol: to begin, an initial ‘pre-treatment’ read was taken just prior to metronidazole (MTZ) treatment. Fish were exposed to MTZ for 24 hours then transferred to control media. A ‘post-treatment’ reading was taken immediately, or on the following day (a 24 hr delay in obtaining post-treatment reads can facilitate clearance of fragmented reporter in some lines). Over the next 4 to12 days, ‘recovery’ readings were taken in order to quantify the return of the targeted cell type. Confocal imaging was used to verify the loss and recovery of nitroreductase (NTR)-targeted cell types (Fig. 3A–D, F–I). The age at which regeneration was evaluated differed for each line; the beta cell regeneration assay was conducted at the late larval to juvenile transition stage (19 dpf to 31 dpf; Fig. 3E), the rod photoreceptor replacement assay at 5 to 11 dpf (Fig. 3J). The data show that ARQiv is capable of detecting the loss and replacement of even small populations of YFP-tagged cell types. . Z-factor (not Z') calculations were used to determine whether ARQiv detection of cell degeneration and regeneration was compatible with HTS (see Materials and Methods for explanation of modifications to this analysis used to evaluate quality of detection of reporter changes in individual over time). The analysis showed that the quality of detecting rod and beta cell loss was very good, 0.48 (±0.11 sem) and 0.47 (±0.07 sem), respectively. However, detection of cell regeneration was not as robust. Despite attaining statistical significance in pair-wise tests (Fig. 3E and 3J), Z-factors at the latest time point of recovery analyzed for rod and beta cells attained values of only −0.68 (±0.6 sem) and −4.01 (±0.15 sem), respectively. Still, any compound which can improve regeneration over the time frame evaluated would produce a stronger Z-factor as the extent of regeneration increased. Thus pursuing HTS for compounds which promote better regeneration remains feasible, particularly for binary assays such as stimulating regeneration in non-regenerative mutants.

### Quantification of Genetic and Chemical Modulation of the Notch Signaling Pathway

**Figure 4:**
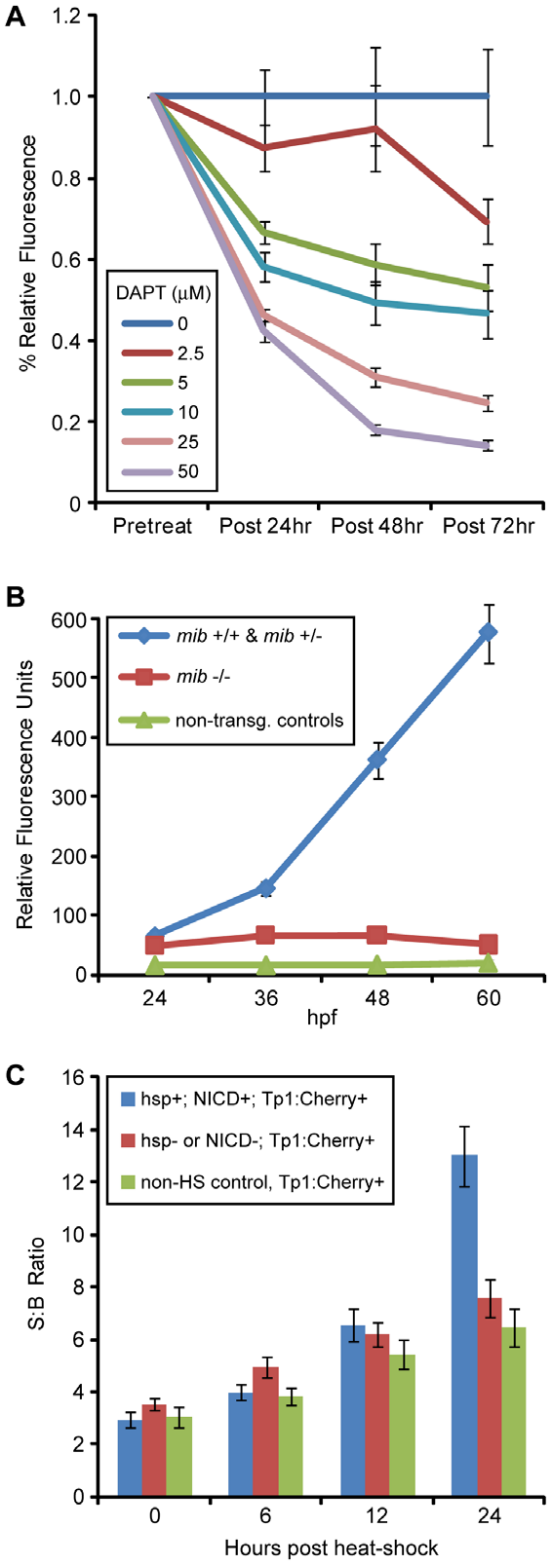
ARQiv detection of modulations in Notch pathway signaling. A) Comparison of fluorescence levels in the Notch-signaling transgenic reporter line, *Tp1:GFP* treated with increasing concentrations of DAPT (an inhibitor of gamma-secretase proteolytic activity). Following pre-treatment scans at 48 hpf, DAPT treatments commenced and post-treatment scans were taken every 24 hrs thereafter until 120 hpf. The results show a concomitant decrease in Notch signaling with increasing DAPT concentration. Paired t-tests showed that all DAPT treatments of 5 µM and above are significantly different (p-value ≤0.05) than controls from post-24 hr onward. B) Comparison of fluorescence levels in *Tp1:Cherry* embryos within the context of the Notch pathway mutant *mindbomb (mib).* The *mib* mutation is believed to abrogate Notch-signaling by preventing normal function of the ligand Delta. Embryos from a *mib*/+; *Tp1:Cherry* incross were phenotypically screened at 24hpf to delineate *mib* (-/-), from wildtype (wt, +/+) and heterozygotes (het, +/-), as well as to identify non-transgenic siblings. Reporter expression levels were then quantified every 12 hrs thereafter until 60 hpf. The wt transgenic embryos show a steady increase in Notch reporter expression; however, the *mib* transgenic embryos show a constant low expression level near that of non-transgenic controls. Paired t-tests showed revealed the *mib* embryos produced significantly lower (p-value ≤0.05) reporter levels than wt and het siblings starting at 24 hpf. C) Comparison of fluorescence levels in *Tp1:Cherry* embryos within the context of Notch pathway activation following induction of NICD expression in *Tp1:Cherry*; *hsp:Gal4; UAS:NICD* triple transgenic larvae. Controls included both non-heat shocked *Tp1:Cherry* ^+^ siblings and heat-shocked *Tp1:Cherry* ^+^ siblings in which *hsp:Gal4* and/or *UAS:NICD* was not present. Prior to heat shock a pre-treatment scan was performed at 4 dpf. An initial heat shock was performed to induce expression of NICD and repeated 12 hrs later. Scans were performed every 6 hrs over the next 24 hrs. Larvae with a slope of mCherry expression two standard deviations above non-heat shock controls were considered positive for all three transgenes and pooled. Larvae below this cut-off were considered to be negative for at least one of the other transgenes and pooled as heat-shocked negative controls. The data show that over-expression of NICD leads to a significant increase in expression of the mCherry reporter at 24 hours (p-value = 8.3E^−06^) post heat-shock in keeping with ligand-independent activation of the Notch pathway.

To determine whether ARQiv could be used to detect changes in relative levels of a molecular signaling pathway, Notch reporter lines *Tg(Tp1glob:EGFP)^um14^* and *Tg(Tp1glob:hmgb1-mCherry*)*^jh11^* (hereafter, *Tp1:GFP* and *Tp1:Cherry*, respectively, [31] were subjected to chemical and genetic modulations. The Notch pathway inhibitor N-[N-(3,5-Difluorophenacetyl)-L-alanyl]-S-phenylglycine t-butyl ester (DAPT) [40] was tested across a range of concentrations used in prior zebrafish publications (up to 100 µM). Following a pre-treatment scan at 48 hpf, *Tp1:GFP* larvae were exposed to DAPT at the indicated concentrations over the next 3 days (with DAPT refreshed daily). Post-treatment scans were performed every 24 hrs over the course of the treatment (72, 96, and 120 hpf). The results show a clear dose-dependent change in reporter fluorescence over time (Fig. 4A). Similarly, we tested the effects of the Notch pathway mutant *mindbomb* (*mib*) on Tp1-dependent reporter expression using the *Tp1:Cherry* line. Larvae from a cross between *Tp1:Cherry*; *mib/+* fish were visually phenotyped into mutant, wildtype or heterozygous, and non-transgenic pools at 24 hpf. Scans were then performed at 24, 36, 48, and 60 hpf. The results show that *mib* mutant larvae show statistically significant deficits in reporter gene expression compared to wildtype and *mib* heterozygote siblings (Fig. 4B). Activation of the Notch pathway induces a proteolytic cascade, culminating in production of the Notch intracellular domain (NICD) which acts as a transcriptional activator [41]. To hyperactivate the Notch pathway, *Tp1:Cherry* fish were crossed with a double transgenic line wherein expression of NICD is induced upon heat shock (i.e., *hsp70l:Gal4; UAS:notch1a-intra*) to produce *Tp1:Cherry*; *hsp:Gal4*; *UAS:NICD* triple transgenic progeny. A pre-treatment scan was taken at 4 dpf, larvae were then heat shocked by incubating at 39°C for one hour, after which fish were returned to 28.5°C (a second heat shock treatment was performed 12 hrs later). Post-treatment reads were taken at 6, 12, and 24 hrs post initial heat shock and fish producing signal two standard deviations above non-heat shock controls were considered to be positive for all three transgenes – in keeping with prior correlations between visually detectable reporter expression and genotyping results [31]. The results indicate that hyperactivation of the Notch pathway can also be detected by ARQiv analysis (Fig. 4C).

### Quantification of Reactive Oxygen Species Accumulation

**Figure 5:**
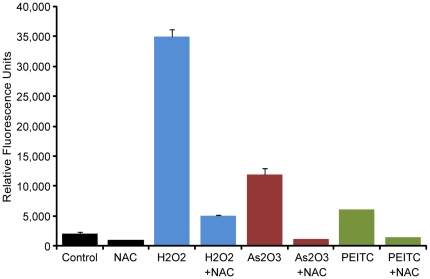
ARQiv detection of accumulated reactive oxygen species (ROS). Comparison of fluorescent dye levels in wildtype DCFH-DA-treated zebrafish embryos exposed to the indicated agents for a total of 30 min exposure time. Results show significant increases in DCFH-DA fluorescence following exposure to agents known to induce oxidative stress. Treatment with the ROS scavenger NAC reduces fluorescence (see text for further details). Pair-wise t-test comparisons between agent-treated and agent + NAC-treated subjects resulted in the following p-values: H_2_O_2_ = 3.8E^−10^, As_2_O_3_ = 5.5E^−07^, PEITC = 9.8E^−05^. Abbreviations: NAC, *N*-acetylcysteine, (100 µM); H_2_O_2_, Hydrogen peroxide (1 mM), As_2_O_3_, Arsenic trioxide (10 µM), PEITC, Phenethyl isothiocyanate (5 µM).

Finally, we tested the ability of ARQiv to detect changes in physiological state using 2′, 7′-dichlorodihydrofluorescein-diacetate (DCFH-DA) to quantify accumulation of reactive oxygen species (ROS) following exposure to oxidative stressors. Prior studies had shown a similar approach [using 5-(and-6)-chloromethyl-2¶,7¶-dihydrodichlorofluoresceindiacetate (CM-H_2_DCFA)] was viable for detecting a ∼2.5-fold increase in ROS following exposure of zebrafish embryos to ionizing radiation [19]. DCFH-DA works by oxidation-dependent conversion of the non-fluorescent 2′,7′-dichlorodihydrofluorescein (DCFH_2_) to the fluorescent 2′,7′-dichlorofluorescein (DCF) [42]; fluorescence thus increases relative to ROS production. 4 hpf embryos were pre-treated with either the ROS scavenging compound, *N*-acetylcysteine (NAC, 100 µM), or vehicle control for 2 hrs, then exposed for 30 min to the following known oxidative stressors: hydrogen peroxide (H_2_O_2_, 1 mM), arsenic trioxide (As_2_O_3_, 10 µM), phenethyl isothiocyanate (PEITC, 5 µM). Immediately following the treatments above, DCFH-DA (50 µM) was added for 1 hr and post-treatment scans were taken. The results show that when DCFH-DA-treated embryos are exposed to agents known to increase oxidative stress, ARQiv can detect concomitant increases in DCF fluorescence indicating accumulation of ROS (Fig. 5). *N*-acetylcysteine treated controls and co-treated embryos show reductions in fluorescence consistent with NAC-mediated ROS scavenging.

## Discussion

Methods for rapidly collecting phenotypic data facilitate large-scale genetic and chemical screens in living animal models. An innovative array of “high-content” *in vivo* imaging platforms exist but many are either not widely available [4] or are specific to a given assay [11], [43], [44], [45]. Importantly, nearly all whole-organism screening methods that have been developed remain limited to mid-throughput volumes (i.e., ∼5,000 units per day) due to the time required for image acquisition and/or image processing stages. We therefore sought to establish a simplified quantitative method for monitoring reporter signals *in vivo* at true HTS volumes (i.e., ≥50,000 units per day). We reasoned that established quantitative HTS assay systems might provide a viable solution and explored the use of microplate readers for quantifying reporter changes in living zebrafish.

The ARQiv system is a simple and versatile method for quantifying transgenic reporter expression levels in individual zebrafish embryos, larvae, and juveniles. ARQiv complements automated high-content imaging methods by promoting a tiered approach to *in vivo* compound screening; ARQiv serving as an initial high-throughput step for identifying compound candidates followed by high-content screens to reveal visual details. One of the main advantages of this system is the ability to monitor reporter expression changes over time in individuals, thus allowing internal normalization of relative expression levels. Other advantages of this system, relative to other whole-organism screening systems include, 1) Increased throughput –true HTS volumes, and 2) Accessibility – economical off-the-shelf instrumentation.

As a demonstration, we performed assays for cellular loss and regeneration, manipulation of the Notch signaling pathway, and accumulation of reactive oxygen species (ROS). The data demonstrate that ARQiv was sensitive enough to detect the loss and replacement of fluorescently labeled cells (Fig. 3). Importantly, the loss and regeneration of even small numbers of cells can be detected. This is particularly evident in the pancreatic beta cell regeneration assay (Fig. 3A–E). ARQiv was also capable of monitoring chemical (Fig. 4A) and genetic inhibition (Fig. 4B), as well as activation (Fig. 4C) of a Notch reporter over several days. Finally, a dye that is converted to a fluorescent form by oxidants provided a general measure of ROS accumulation in embryonic (Fig. 5) and larval (data not shown) zebrafish.

The beta cell regeneration paradigm – an inducible Type I diabetes model – demonstrates two other potentially useful aspects of the ARQiv system, 1) ratiometric analyses, and 2) the ability to evaluate fish at later stages of development (19 to 31 dpf). Ratiometric methods, whereby two independent reporter signals are assessed in the same sample, have been used to normalize signals to cell number and are useful for HTS assays [46]. Here, ratiometric scanning was used to monitor changes in PhiYFP-expressing cell numbers (beta cells) relative to neighboring tagRFP-expressing control cells which should remain stable (delta cells). This approach subserves two key functions: 1) ensures that the plate reader scanned the appropriate region of the subject (particularly important when experimental signals can drop to background levels, as is the case in a cell ablation assay), 2) verifies and/or defines the location of the region/tissue of interest (when using multi-regional scans), and 3) provides a means to detect relative changes in cellular compositions of the labeled tissue. For instance, this could facilitate identification of conditions leading to hypo- and hyper-regenerative responses.

Although ARQiv attains our goal of establishing a HTS-compatible *in vivo* screening platform, achieving true HTS volumes requires that adequate numbers of fish embryos/larvae can be generated per day and that they can be singly arrayed into multiwell formats. Fortunately, solutions to both these problems are commercially available. For instance, Aquatic Habitats has developed a mass embryo production system that is capable of generating the numbers of eggs per day required for HTS. In addition, Union Biometrica markets a Complex Object Parametric Analysis and Sorting (COPAS™) instrument which functions essentially as a fluorescence activated sorter for small model organisms, capable of sorting for viability and/or fluorescent reporter expression. The ability to sort subjects based on reporter expression levels provides a simple means of reducing variability (i.e., by eliminating low expressing larvae as subjects are arrayed into multiwell formats) and thereby to increase assay quality to HTS-compatible levels. However, the COPAS cannot be used to track changes in expression over time in individuals – a central strength of the ARQiv and vertebrate automated screening technology (VAST) [4] platforms. Our initial tests with the COPAS verify an embryo dispense rate range of 0.9 to 1.25 sec per embryo/larvae – thus, capable of dispensing >50,000 per day. Until instrumentation becomes available that exceeds this rate it would be necessary to employ multiple COPAS instruments to achieve throughputs of >50,000 per day. Even if this limitation were overcome, another potential issue with our throughput projections is the assumption that round the clock screening is relevant and/or reasonable. For optimal performance, this might require treatment regimens to be delivered in a staggered manner such that each subject was evaluated at an equivalent time point. In addition, certain reporters (e.g., signaling pathway reporters) might be susceptible to natural variance with the circadian cycle. Nevertheless, we show here that applying a quantitative screening approach does promote throughput on a scale from 20–70 times faster than automated high content screening technologies.

Significant individual variance of fluorescent reporter intensities was evident in all of the transgenic lines tested. The source of this variability is unknown but could be the result of several non-exclusive factors, e.g., transgene insertion number [47] and/or epigenetic differences [48]. Regardless, this fact reduces the ability to detect changes in reporter expression if signals are averaged across populations. ARQiv can circumvent this issue by monitoring reporter intensities directly in individual fish over time; an internal normalization approach that allows changes in reporter levels to be more accurately compared across conditions as a percentage of an initial scan. We determined that fluorophores in the 525 nm emission range and above (e.g., YFP, RFP) provide HTS-compatible signal detection when expressed in discrete cell-specific domains in embryonic to juvenile stage zebrafish (i.e., Z'-factors ranging from 0.32 to 0.78 for single scans and 0.51 to 0.87 for 2×2 patterned regional scans, data not shown). One issue that remains as a barrier to achieving HTS screening volumes for time-resolved, individually normalized assays, is the ability to rapidly move fish from one experimental condition to another (e.g., anesthetic to standard media). Multiwell insert systems are available for such purposes (e.g., Millipore MultiScreen®-Mesh plates), however to date none have been designed for reporter-based assays (e.g., black polypropylene for fluorescence).

HTS methods were intended to streamline and economize the drug discovery process. However, new chemical entity disclosures and investigational new drug applications have declined over the past decade [49]. It has been proposed that this trend is at least partially due to the absence of complementary *in vivo* HTS methods available for biological validation of lead compounds. Indeed, the time and cost of testing leads in mammalian systems is prohibitively high, relegating this step to later stages of the discovery process where failure rates are high [50]. Zebrafish are being integrated into “functional pharmacology” drug discovery platforms in an effort to address the issue of biological validation at earlier stages of the process. In this regard, ARQiv provides a generally useful screening method, useful for large-scale, time-resolved, reporter-based assays in zebrafish.

It is abundantly clear that human diseases can be successfully modeled in zebrafish [51], [52], [53], [54]. Moreover, and of particular importance here, chemical compounds have been shown to illicit remarkably similar effects in fish and humans [13], [52], [55], [56]. The zebrafish model system is therefore well positioned to provide HTS-ready solutions to drug discovery – a fact that could be significantly augmented by the availability of quantitative reporter-based assays. Accordingly, we have developed an automated, economical, and universally applicable platform for assessing the activity of reporter-based read outs over time in living zebrafish embryos, larvae, or juveniles. Importantly, our analysis indicates that most reporter-based assays developed for zebrafish should be immediately adaptable to the ARQiv platform. Thus, the versatility and ease of deployment of this platform should serve to rapidly expand the kinds of whole-organism HTS assays for which the zebrafish system can be utilized.

## Supporting Information

Figure S1 **Autoflourescence in wildtype and pigmentation mutant zebrafish.** To better define autofluorescent “noise”, three different strains representing different pigmentation patterns, wildtype, *roy orbison* (*roy*) mutants, and *roy orbison;albino* (*roy;alb*) double mutants were scanned from 1 dpf through 5 dpf. These mutants reduce the number of iridiphores (*roy*), or the number of iridiphores and melanophores (*roy;alb*), and were chosen due to the fact that detection of fluorescent reporters is enhanced in these lines at later stages of development, which is of particular relevance to studies of regeneration. In addition, wildtype and *roy* were scanned in the presence and absence of PTU as a further means of determining the relative contribution of melanaphores to autofluorescence issues. Intensity plots of autofluorescence profiles of three different fish strains ±PTU ares shown. X-axis is wavelength of light, y-axis is relative fluorescence intensity units, and outputs are color-coded according to the emission profile tested. To assess autofluorescence, 2D and 3D scans were performed using 5 nm excitation and 5 nm emission bandwidth sweeps for “blue” (ex405/em415-700), ‘cyan’ (ex440/em450-700), ‘green’ (ex488/em498-700), ‘yellow’ (ex515/em525-700), ‘red’ (559/569-700), and infrared fluorophores (635/645-700) – the excitation wavelengths chosen were matched to available confocal laser lines. Autofluorescence intensity increases, particularily in the blue and cyan emission ranges, when fish are rendered “transparent” by chemical (+PTU) or genetic (roy;alb) means. Each trace is the average of 8–12 fish per condition.
(TIF)

Figure S2 **Autoflourescence in adult pigmentation mutants.** Autofluorescence profile of roy orbison;albino (roy;alb) double mutant fish at adulthood. Micrographs were taken on an Olympus SZX16 fluorescence steroscope, shown are composites of transmitted and fluorescence images. Autofluorescence was characterized using filter sets for common fluorescent reporters as listed. Region specific autofluorescence is most evident in the gut, and is particularly strong in the red emission wavelengths.
(TIF)
