# Supplementary figures and images for: Automated Reporter Quantification In Vivo: High-Throughput Screening Method for Reporter-Based Assays in Zebrafish
Source: PLoS One. 2012 Jan 4;7(1):e29916. doi: 10.1371/journal.pone.0029916 (PMC3251595; doi:10.1371/journal.pone.0029916)

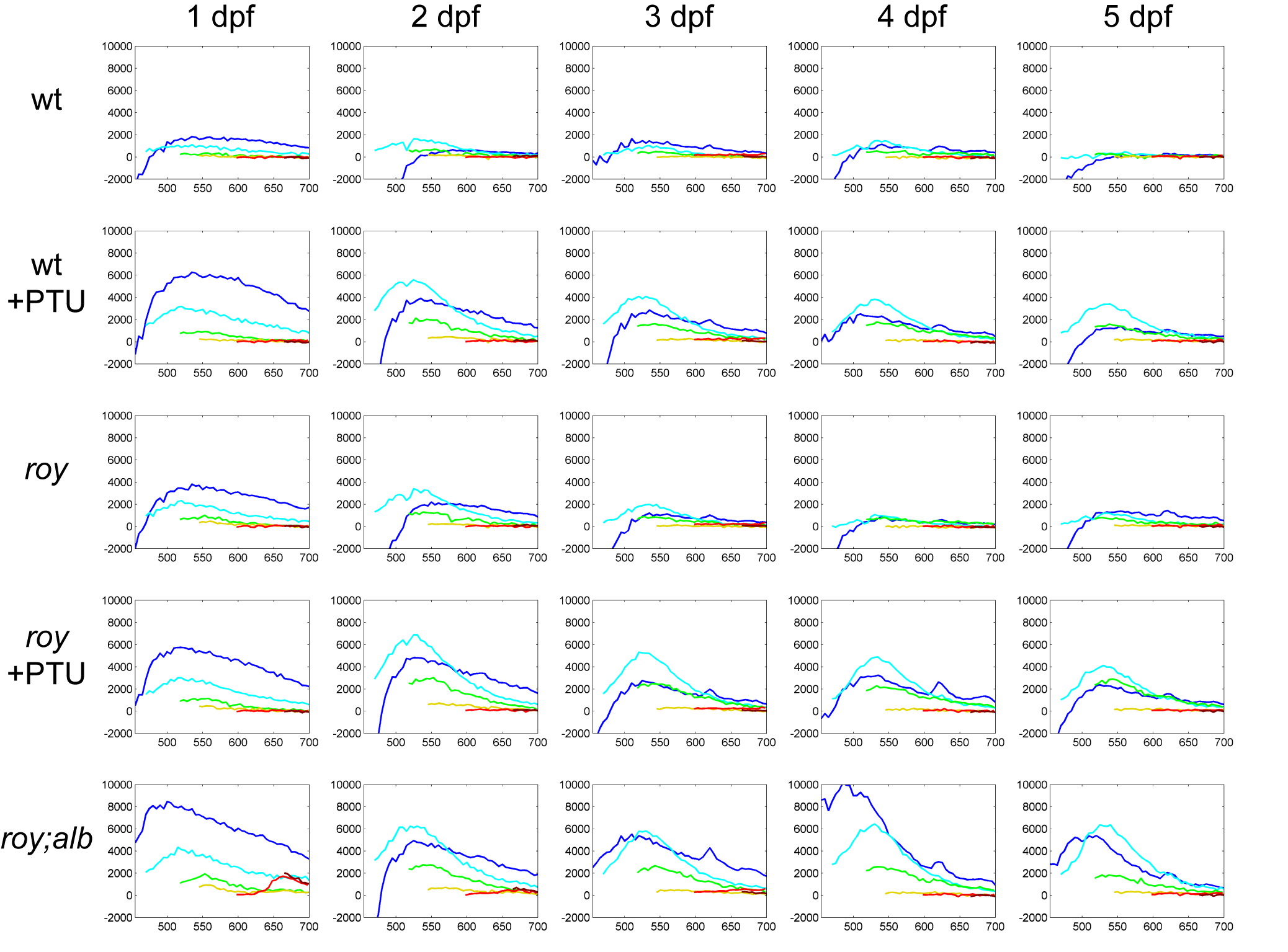

Supplement: Figure S1 — Autoflourescence in wildtype and pigmentation mutant zebrafish. To better define autofluorescent “noise”, three different strains representing different pigmentation patterns, wildtype, roy orbison (roy) mutants, and roy orbison;albino (roy;alb) double mutants were scanned from 1 dpf through 5 dpf. These mutants reduce the number of iridiphores (roy), or the number of iridiphores and melanophores (roy;alb), and were chosen due to the fact that detection of fluorescent reporters is enhanced in these lines at later stages of development, which is of particular relevance to studies of regeneration. In addition, wildtype and roy were scanned in the presence and absence of PTU as a further means of determining the relative contribution of melanaphores to autofluorescence issues. Intensity plots of autofluorescence profiles of three different fish strains ±PTU ares shown. X-axis is wavelength of light, y-axis is relative fluorescence intensity units, and outputs are color-coded according to the emission profile tested. To assess autofluorescence, 2D and 3D scans were performed using 5 nm excitation and 5 nm emission bandwidth sweeps for “blue” (ex405/em415-700), ‘cyan’ (ex440/em450-700), ‘green’ (ex488/em498-700), ‘yellow’ (ex515/em525-700), ‘red’ (559/569-700), and infrared fluorophores (635/645-700) – the excitation wavelengths chosen were matched to available confocal laser lines. Autofluorescence intensity increases, particularily in the blue and cyan emission ranges, when fish are rendered “transparent” by chemical (+PTU) or genetic (roy;alb) means. Each trace is the average of 8–12 fish per condition. (TIF) [file pone.0029916.s001.tif]

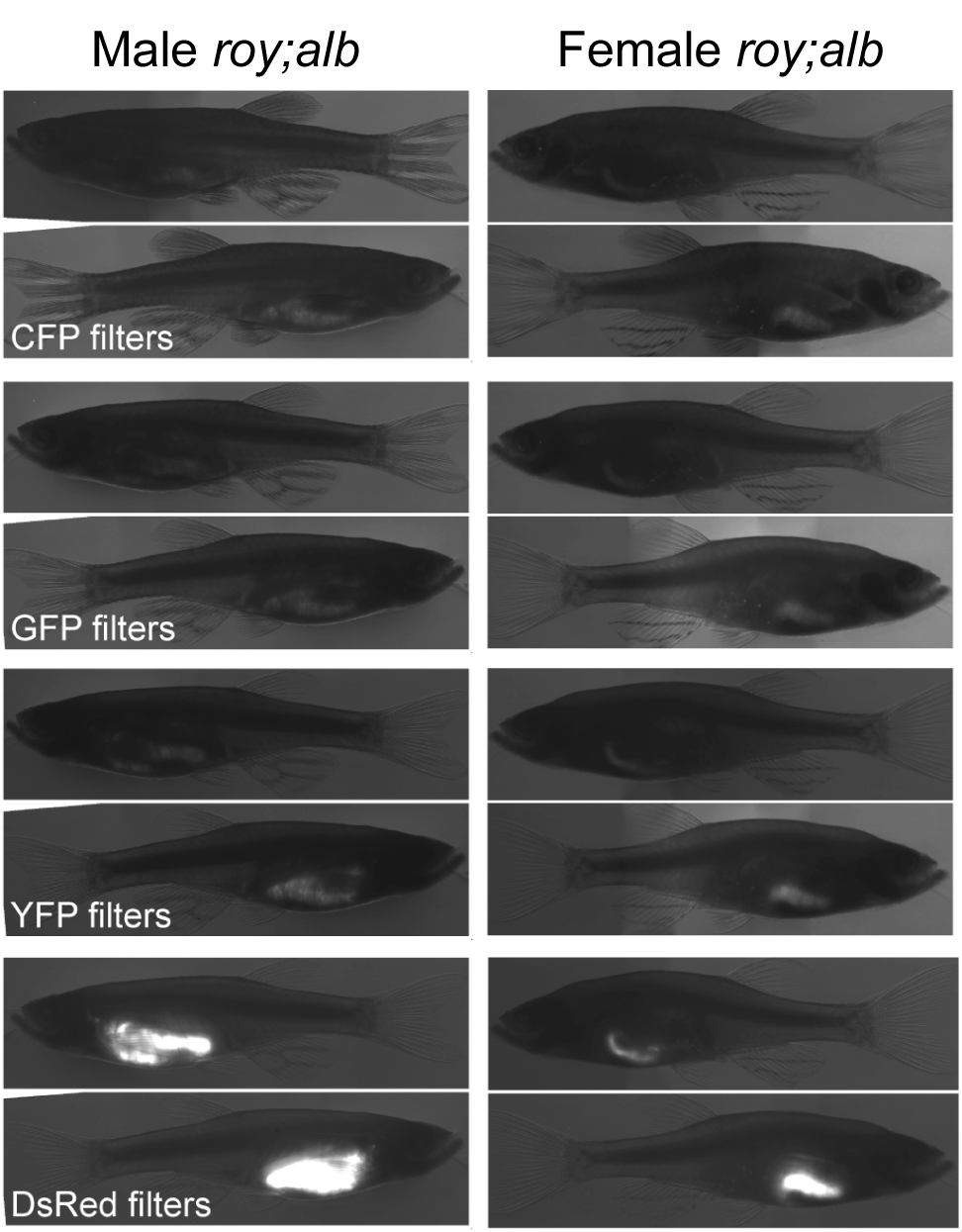

Supplement: Figure S2 — Autoflourescence in adult pigmentation mutants. Autofluorescence profile of roy orbison;albino (roy;alb) double mutant fish at adulthood. Micrographs were taken on an Olympus SZX16 fluorescence steroscope, shown are composites of transmitted and fluorescence images. Autofluorescence was characterized using filter sets for common fluorescent reporters as listed. Region specific autofluorescence is most evident in the gut, and is particularly strong in the red emission wavelengths. (TIF) [file pone.0029916.s002.tif]
